# Numerical Study of Incidence Angle-Tuned, Guided-Mode Resonant, Metasurfaces-Based Sensors for Glucose and Blood-Related Analytes Detection

**DOI:** 10.3390/s25185852

**Published:** 2025-09-19

**Authors:** Zeev Fradkin, Maxim Piscklich, Moshe Zohar, Mark Auslender

**Affiliations:** 1 Department of Electrical and Electronics Engineering, Sami Shamoon College of Engineering, Ashdod 7724503, Israel; zeevfr@ac.sce.ac.il (Z.F.);; 2 Department of Electrical and Electronics Engineering, Sami Shamoon College of Engineering, Beer-Sheva 8410802, Israel; 3 School of Electrical and Computer Engineering, Ben-Gurion University of the Negev, P.O. Box 653, Beer-Sheva 8410501, Israel

**Keywords:** guided-mode resonance, optical gratings, polymers, cesium-lead halide perovskite

## Abstract

In optical one-dimensional grating-on-layer planar structures, an optical resonance occurs when the incident light wave becomes phase-matched to a leaky waveguide mode excited in the layer underneath the grating by an appropriate tuning of the grating periodicity. Changing the refractive indices of the grating’s constituents, and/or thickness, changes the resonance frequency. In the case of a two-dimensional grating atop such a smooth layer, a similar and also cavity-mode resonance can occur. This idea has straightforward usage in diverse optical sensor applications. In this study, a novel guided-mode resonance sensor design for detecting glucose and hemoglobin in minute concentrations at a wide range of incidence angles is presented. In this design, materials of the grating, such as a polymer and cesium-lead halide with a perovskite crystal structure, are examined, which will allow flexible, low-cost fabrication by soft-lithography/imprint-lithography methods. The sensitivity, figure of merit, and quality factor are reported for one- and two-dimensional grating structures. The simulations performed are based on rigorous coupled-wave analysis. Optical resonance quality factor of ∼$5\cdot 10^{5}$ is achieved at oblique incidence for a structure comprising a one-dimensional grating etched in a poly-vinylidene chloride layer atop a silicon nitride waveguide layer on a substrate. Record values of the above-noted characteristics are achieved with a synergetic interplay of the materials, structural dimensions, incidence angle, polarization, and grating geometry.

## 1. Introduction

The guided-mode resonance (GMR) effect has great potential in different applications such as sensors [1], optical filters [2], and solar cells [3]. A basic GMR structure includes an optical diffraction grating and a smooth dielectric waveguide layer underneath. Under resonance conditions, a sharp reflection peak is monitored on the wavelength axis due to the excitation of the waveguide leaky mode by diffraction of incident light into the waveguide [4]. Different GMR structures for sensitivity-enhanced sensing were published, including the use of dielectric double-layer [5] or multilayer [6] structures, dielectric nanorods [7], and metal layers [8]. These structures require complicated fabrication processes.

Our approach possesses the following two key advantages for fabricating and operating the GMR sensors compared to the conventional approach, which often prescribes high-cost photolithography, and operation at normal incidence resulting in limited quality factors (Q-factors)—*Q*, see definition for further details—typically in the range of ∼100–300 [1]. First, the proposed structures are compatible with soft and nanoimprint lithography, enabling low-cost, large-area fabrication with no need for complex photolithography processes [9,10]. Second, operating the sensor under oblique incidence substantially improves the *Q* and figure of merit (FOM)—see definition for further details—performance markers consistently with the recent findings, that oblique incidence can enhance sensitivity by up to ∼2.9 times due to a synergy of grating and waveguide sensitivities [11].

The objection to using oblique incidence in favor of a normal one in reflectance measurements for certain applications is often argued by the polarization independence, ease of alignment, and simpler setups for normal incidence. Also, due to a need for precise angle monitoring with more complex equipment, oblique incidence measurements seem technically more challenging. But the following should be taken into account: in the normal incidence case, the reflected wave is to be diverted away from the normal to avoid interference with the incident one, which needs a special setup and results in measured intensity loss; the polarization independence breaks down for the structures embedding gratings. These details, and also the fact that even for plane-parallel surfaces, the oblique incidence can provide higher reflectance and more sensitive and accurate information, making implementing it worthwhile. Thus, the oblique incidence paradigm and the above-noted advantages make our design highly promising for practical, scalable, and cost-effective sensing applications.

This work presents GMR sensor designs based on sub-wavelength grating-on-waveguide structures with grating mastered in cesium-lead tri-bromide (CsPbBr_3_) crystal with perovskite structure, and poly-vinylidene chloride (PVC). These sensors will allow low-cost soft lithography fabrication methods, such as imprint nanolithography [12], dip-pen nanolithography, and others, without expensive photolithography equipment and fabrication. The feasibility of fabricating the proposed structures using soft lithography methods should also be emphasized. Techniques such as nanoimprint lithography and replica molding enable low-cost, high-resolution patterning without needing cleanroom facilities. These methods are particularly suitable for polymeric and perovskite materials, and have been successfully demonstrated for metasurface fabrication using reusable PDMS molds [13]. Furthermore, Huang et al. [14] reported a one-step sol–gel imprint lithography process for fabricating the grating and waveguide layers of GMR structures in a single step, offering a rapid and scalable route for device production. In recent years, high refractive index polymers [15] and lead-halide perovskites have been extensively researched due to the possibility of flexible, low-cost fabrication and their excellent optical properties for solar cells and other photonic devices [16].

The lead-halide perovskites are emerging as promising materials for nanoscience and nanotechnology and can be used in various applications from detectors to lasers. Extensive studies of nano-lasers and nano-photodetectors have been reported [17]. Recently, CsPbBr_3_-waveguide prototypes were fabricated [18,19]. The fabrication of the perovskite nano-photonic structures, such as nanoscale metasurfaces, is demonstrated via soft lithography, a method in which the patterning is performed when the perovskite material is not fully crystallized, allowing for crystallization within the mold with the end result of facile and unharmful imprinting of sub-micron features onto perovskite thin films [13].

Cefarin et al. [20] demonstrated a nanostructured perovskite layer patterning process using pulsed nanoimprint lithography (NIL). Polymers also play an important role in photonic materials. Several research studies have shown the application of soft lithography methods for fabricating polymeric photonic devices. Polymers are relatively inexpensive and used as materials for photonic applications in several ways [21].

Perovskite and silicon nitride were selected as sensor materials for two key reasons. First, to validate that slight differences in material parameters—such as refractive index—have a measurable but limited impact on sensor performance, consistent with prior modeling studies [22]. Second, it demonstrates the flexibility of the design, which allows choosing between materials based on fabrication compatibility and process preferences. This flexibility is particularly relevant for scalable and cost-effective manufacturing, as supported by recent integration studies [23,24].

## 2. Materials and Methods

### 2.1. Analyzed Sensors’ Structures and Their Characteristics

**Figure 1:**
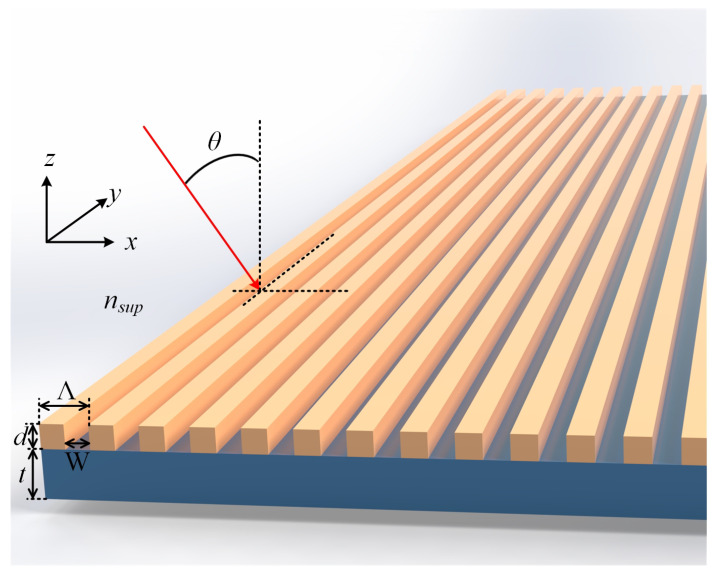
Schematic of 1D grating-on-waveguide structure-based sensor. The dimensional parameters of the structure shown are: the grating period (pitch) Λ, grating line (lamella) height *d*, grating slot (groove) width *W*, and the waveguide layer thickness *t*.

**Figure 2:**
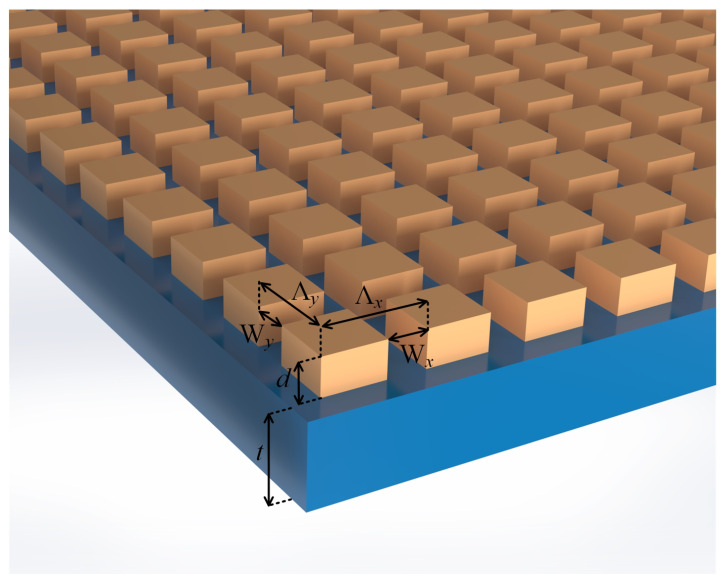
Schematic of 2D grating-on-waveguide structure-based sensor. The dimensional parameters of the structure shown are as follows: the grating pitches in the lateral *x*- and *y*-directions $\Lambda_{x}$ and $\Lambda_{y}$, respectively, corresponding to grating slot widths $W_{x}$ and $W_{y}$, grating mesa height *d*, and the waveguide layer thickness *t*.

The structures of the grating-based sensors for the 1D- and 2D-grating cases are presented in Figure 1 and Figure 2, respectively. The sensors include the correspondingly patterned gratings, with rectangular cross-section lines (Figure 1) and cuboid mesas (Figure 2), and optical waveguides beneath. The structures are assumed to be fabricated on a substrate with a refractive index (RI) of $n_{\mathrm{sub}}$. A tested liquid material (assay or analyte), the ambient structures from above, has a RI of $n_{\sup}$. For a resonant reflection response sensitive to $n_{\sup}$, a linearly polarized plane wave light is directed to the structures under an incidence angle θ at the *x*-*z* incidence plane (the *z*-axis is normal to the structures’ surface).

Instructive dimensionless parameters for the structures shown in Figure 1 and Figure 2 are the so-called duty cycles (DCs). In the 1D case, DC=1−W/Λ, whereas two DCs can be defined in the 2D case, viz., ${\mathrm{DC}}_{x}=1-W_{x}/\Lambda_{x}$ and ${\mathrm{DC}}_{y}=1-W_{y}/\Lambda_{y}$. The considered grating materials are silicon nitride (Si_3_N_4_), PVC, and above-noted CsPbBr_3_, whereas the waveguide materials are either Si_3_N_4_ or CsPbBr_3_, and the substrate is the soda–lime glass throughout. For the DC parameters, we, in advance, chose constant values, namely, $\mathrm{DC}={\mathrm{DC}}_{x}={\mathrm{DC}}_{y}=0.5$.

We simulated the spectra of reflectance (normalized reflection efficiency) R(λ) for the GMR sensors in question using a full-vector Fourier series expansion-based technique, most often called the rigorous coupled-wave analysis (RCWA). This semi-analytical method of electromagnetic (EM) computations is applied to solving problems of scattering from periodic structures, which was first developed by Knop [25] and later, dubbed RCWA, independently by Moharam and Gaylord [26,27,28]. In detail, RCWA employs the Fourier expansion of the sought EM fields with respect to lateral coordinate(s) *x* (x,y), which transforms the Maxwell equations in spatial coordinates into ordinary differential equations in the vertical coordinate *z* for infinite-dimensional vectors built from the Fourier components of the fields. For numerical solution, these equations should be truncated by retaining a finite number *N* of the Fourier components. When the structure under study is stacked from periodically patterned or smooth layers, the in-layer partial solutions are matched at the layers’ interfaces according to celebrated EM boundary conditions.

Still, despite the ease of implementation and widespread use of the initial RCWA since the start of the 1980s, strong evidence of numerical instability upon increasing the grating depth, number of layers (or both), and severe slow-convergence problems with increasing *N*, were reported throughout the 1990s; see, e.g., [29,30,31,32] and [33], respectively, to mention a few studies. Since then, RCWA has been recast to achieve improved convergence [34,35,36] and numerical stability [31,32,36,37]. Our in-house code of RCWA includes the following: (i) Fourier-factorization recast due to numerically and mathematically based inverse Laurent rules for 1D and 2D gratings [34,35,36,38,39], which provides the truncation error of $O(N^{-a}),\;a≃3$; (ii) an in-layer scattering matrix propagation algorithm [31,36] ensuring unconditional numerical stability; (iii) the truncation error control with combination of Richardson and Aitken extrapolations to the limit. The simulations of structures under study with our code achieve moduli of the absolute error in R(λ) below $0.5\cdot 10^{-3}$ starting at N=35.

**Figure 3:**
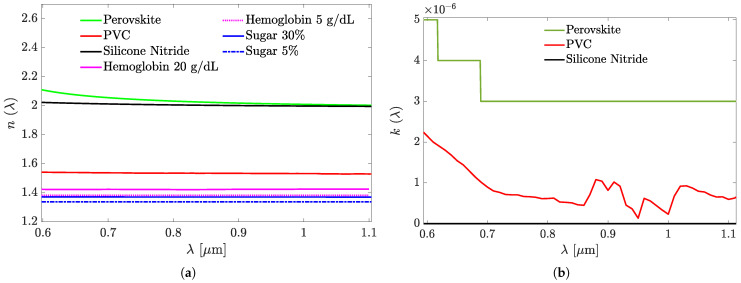
The refractive indexes’ (**a**) and extinction coefficients’ (**b**) spectra of the materials used for the studied sensor structures designs. The details are shown in the graphs’ legends.

For their use in the simulations, the spectra of the RIs n(λ) and extinction coefficients k(λ) were compiled from the published data for the CsPbBr_3_ [40], PVC [41], Si_3_N_4_ [42], hemoglobin [43], and sugar [44], and visualized as graphs in Figure 3. Figure 3b shows the extinction coefficient k(λ) values, which are negligible and therefore do not affect the simulation results. For materials not included in Figure 3b, k(λ) was assumed to be zero. Note that the black line in Figure 3b represents the k(λ) values of Silicon Nitride, which are also approximately zero. The conversion from hemoglobin concentration (g/dL) to refractive index was based on the model proposed by Friebel and Meinke [43]. As is customary for the GMR structures [2], and will also be shown for those considered in what follows: R(λ) has a sharp, resonant peak at some wavelength $\lambda_{r}$. If the peak is characterized by its full width at half maximum (FWHM), then the resonance sharpness can be described by the resonance *Q*, defined by $Q=\lambda_{r}/\mathrm{FWHM}$. By itself, *Q* has nothing to do with the ability of the GMR structure to sense its ambient material change. Rather, an appropriate direct measure to this end is the sensitivity, defined as $S=\partial \lambda_{r}/\partial n_{\sup}\approx \Delta \lambda_{r}/\Delta n_{\sup}$, where $\Delta n_{\sup}$ is a sufficiently small change in the ambient RI due to a change in the assay concentration, which causes a shift $\Delta \lambda_{r}$ in $\lambda_{r}$.

The *S* value shows the rate at which the resonant wavelength of a sensor shifts, but it does not indicate the wavelength shift that can be resolved. One of the benchmarks on which the resolution essentially depends is the *Q*, sometimes called finesse; see, e.g., [45]. Another benchmark is the wavelength resolution δλ of the reflection measurement setup, which is an indispensable part of the sensor device. The parameter that combines the impacts of the sensitivity and *Q* is the FOM, which is their product FOM=S·Q. The other is the refraction-index limit of detection (LOD), which can be defined as LOD=δλ/S, although another S has been proposed [46].

### 2.2. The 1D- and 2D-Grating-Based Sensor Structures Simulated, Set A and B, Respectively

Two sets of the sensor structures, A and B, were analyzed, which include five and three structures of the types shown in Figure 1 and Figure 2, respectively. All structures under consideration have the same values of d=55nm and t=110nm. In set B, all structures have equal pitches in *x*- and *y*-directions, as seen in Figure 2, with the same values of $\Lambda_{x}=\Lambda_{y}=450\;\mathrm{nm}$. The materials of the lamellae (set A) and mesas (set B) were Si_3_N_4_, CsPbBr_3_, and PVC, and the materials of the waveguide layer the material was Si_3_N_4_. However, for set A, we also tested two combinations of CsPbBr_3_ as the waveguide material with CsPbBr_3_ and PVC grating lines. In more detail, the following structures were considered:

Set A

A1 The grating lamella and waveguide are both CsPbBr_3_;
A2 The grating lamella and waveguide are both Si_3_N_4_;
A3 The grating lamella is CsPbBr_3_ and the waveguide is Si_3_N_4_;
A4 The grating lamella is PVC, and the waveguide is CsPbBr_3_;
A5 The grating lamella is PVC, and the waveguide is Si_3_N_4_.

The grating pitches in the structures A1–A3 and A4–A5 are Λ=450nm and Λ=375nm, respectively.

Set B

B1 The grating mesa and waveguide are both Si_3_N_4_;
B2 The grating mesa is CsPbBr_3_ and the waveguide is Si_3_N_4_;
B3 The grating mesa is PVC and the waveguide is Si_3_N_4_.

## 3. Results and Analysis

### 3.1. Reflectance Spectra and Characteristics of the Set A Sensors

**Figure 4:**
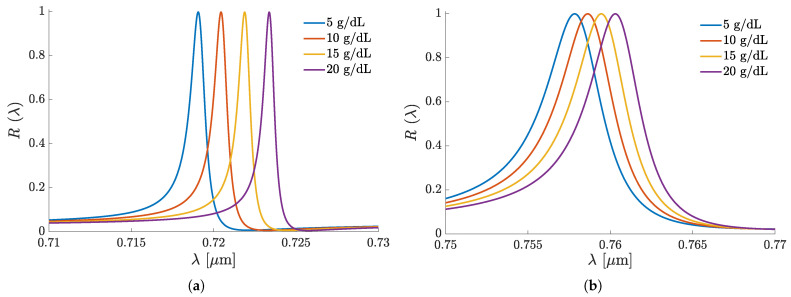
The normal-incidence reflectance spectra of the GMR sensor A1 at different hemoglobin concentrations in water solution: (**a**) p-polarized; (**b**) s-polarized.

The normal-incidence, linearly polarized R(λ) spectra of the structure A1 at varying hemoglobin–water solution assay concentrations are shown in Figure 4. At the s-polarization, also called transverse electric (TE) one, the electric-field vector E remains normal to the incidence plane, i.e., parallel to the grating lines/grooves at all θ. In contrast, at the p-polarization, E lies on the incidence plane, becoming perpendicular to the grating lines/grooves at $\theta =0^{\circ }$. Since at the p-polarization, the magnetic-field vector remains normal to the incidence plane at all θ, it is also called transverse magnetic (TM) polarization.

For both polarizations, as expected, Figure 4 shows resonant reflection spectral lines whose peaks are sensitive to the superstrate water–hemoglobin solution concentration. Yet, the p-polarized reflectance spectrum is notably sharper, i.e., has a higher *Q*, than the s-polarized one. For enlightening explanations, we refer, e.g., to the reviews [2,3,4,46]. The reflection spectra of the other set A sensors are similar in form and peak shifting in response to assay concentration variations, though each has specificity, which will be noted further.

**Figure 5:**
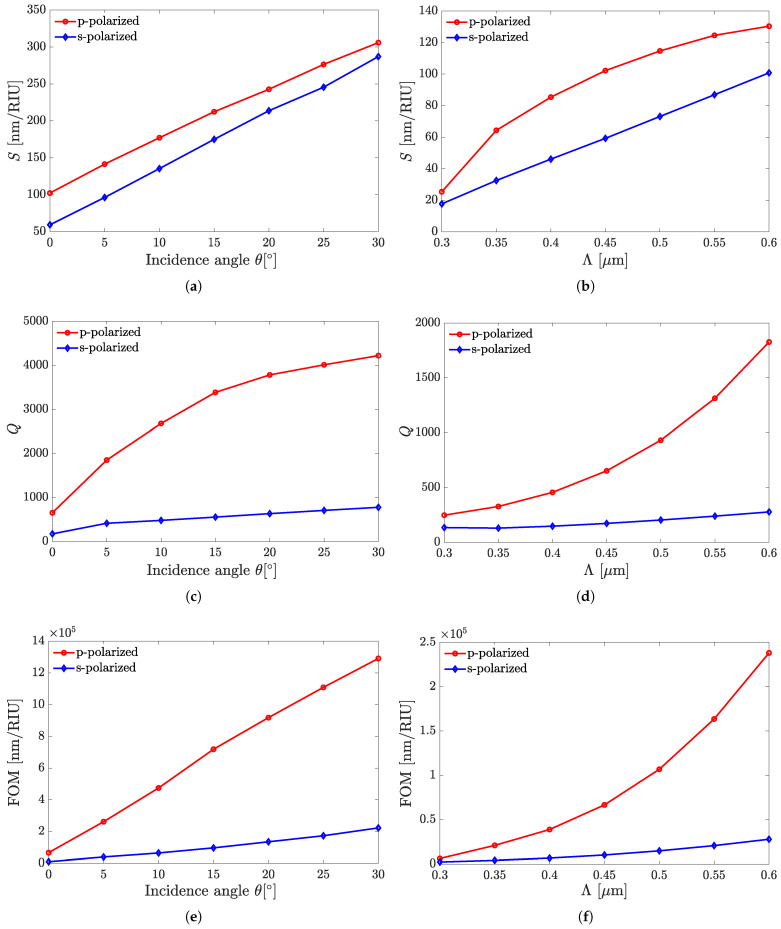
The benchmark characteristics of sensor A1 versus the incident-radiation parameters: angle and polarization (**a**,**c**,**e**); and the grating period at θ=0° (**b**,**d**,**f**).

**Figure 6:**
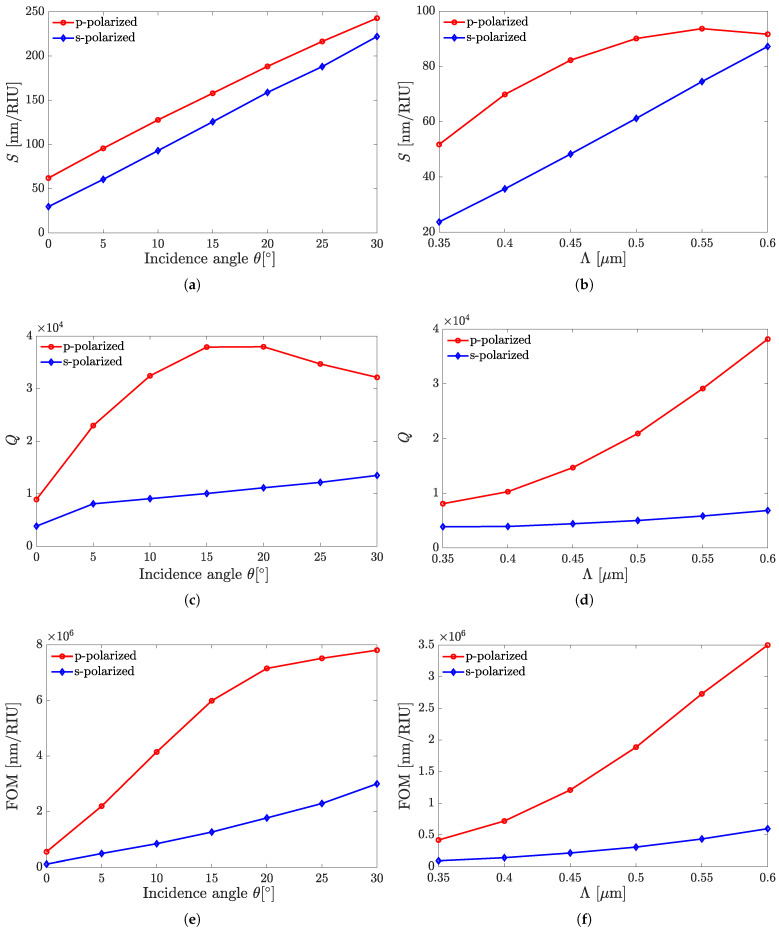
The benchmark characteristics of sensor A4 versus the incident-radiation parameters: angle and polarization (**a**,**c**,**e**); and the grating period at θ=0° (**b**,**d**,**f**).

Here, we present the performance variation for two sensor structures of this kind, namely, A1 and A4, with the incidence angle and grating pitch variations. The previously outlined sensors’ characteristics, such as *S*, *Q*, and FOM, calculated against varying θ and Λ values are shown in Figure 5 and Figure 6 for structures A1 and A4, respectively.

**Figure 7:**
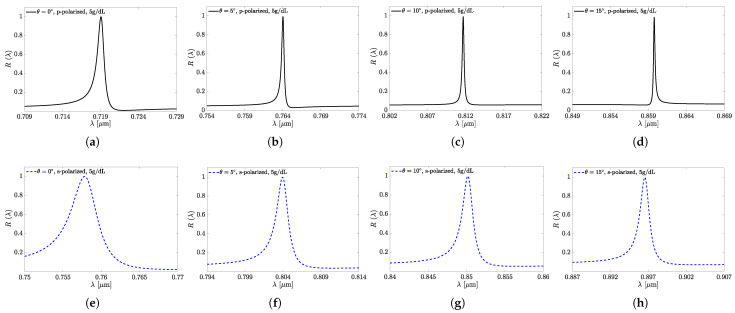
The transformation of the reflection spectra at s-polarization (**a**–**d**) and p-polarization (**e**–**h**) from sensor A1. The incidence angles are as follows: 0° (**a**,**e**); 5° (**b**,**f**); 10° (**c**,**g**); 15° (**d**,**h**).

**Figure 8:**
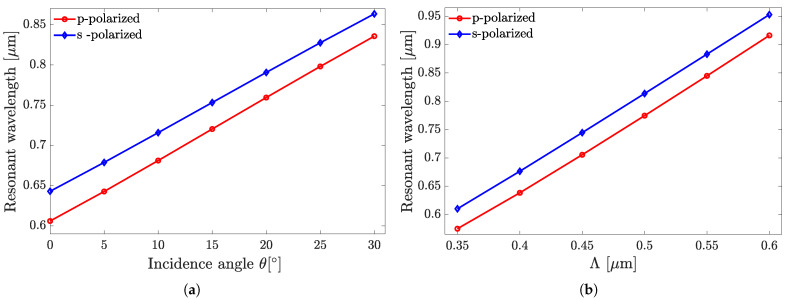
The influence of the angle of incidence (**a**) and grating pitch (**b**) on the resonance wavelength $\lambda_{r}$ of the PVC grating on Si_3_N_4_ waveguide sensor structure (A5). The assay is hemoglobin with a concentration of 5g/dL.

The dependences shown in Figure 5 and Figure 6 demonstrate the sensors’ superiority in all the above benchmarks when performing in the p-polarization mode. Figure 7 illustrates the reflection as a function of wavelength from the all-perovskite sensor at various incidence angles. As the angle of incidence increases, the FWHM decreases more rapidly than the resonant wavelength shifts. Consequently, a larger incidence angle leads to an increase in both the *Q* and the FOM. Figure 8 presents the dependences of $\lambda_{r}$ on θ and Λ in Figure 8a,b, respectively. In the ranges of the respective parameters considered, these dependencies are close to linear ones, which could provide the fabrication scalability.

**Figure 9:**
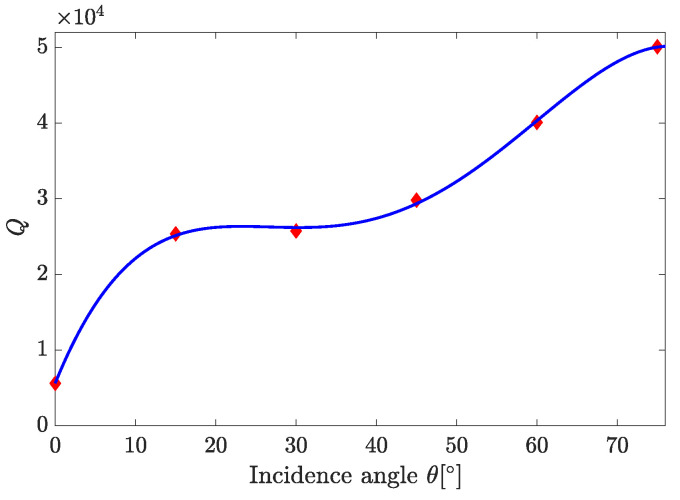
The effect of incidence angle on the *Q* of the A5 sensor structure at p-polarization: small diamonds—computed *Q*; full line—interpolation guide for eye.

Figure 9 presents the dependence of *Q* on θ in the range of $0^{\circ }\leq \theta \leq 75^{\circ }$. It shows that *Q* increases in the range of 0°≤θ≤15° and then, at 15°≤θ≤30°, shows rather weak variations akin to local saturation. Yet, above 30°, an increase in *Q* with increasing θ is observed again, changing from a gradual increase to a steeper one at θ>45°. It is worth noting that the trends that can be concluded from Figure 5, Figure 6, Figure 7, Figure 8 and Figure 9 apply at large, with reasonable reservations, to all sensor structures from set A.

Table 1 summarizes point-wise the benchmark characteristics of all sensor structures from set A versus their types outlined above, as well as the incidence angle, tested for both s- and p-polarizations. The table digitally adds the relevant data for those type A sensors whose characteristics are not covered by Figure 5, Figure 6, Figure 7, Figure 8 and Figure 9. The shown values of *S* and *Q* are after rounding to, at most, three significant digits in the mantissa, while also showing FOM after rounding the product of those two, taken initially at a higher precision. Further, several phenomenological notes concerning the regularities observed in Table 1 are in order. The observations for each of the given structure type are as follows: (i) *S* notably increases with increasing θ, but the increase in the range 0°<θ≤30° is steeper than at 30°<θ≤60°. (ii) *Q* also increases at 0°<θ≤30° at about similar rate as *S* does. In the range 30°<θ≤60°, however, the behavior of *Q* is different at s- and p-polarizations. For the former, it shows a decrease, which is minute for A2 and A3, and drastic for A5. For the latter, *Q* continues to notably increase, as seen in Figure 9. (iii) FOM, contrary to *Q*, increases with increasing θ throughout the simulated range for both polarizations at about the same rate. While in the range 0°–30°, the increase is slightly more than the order of magnitude, it amounts to only a few times at 30°<θ≤60°. (iv) *S* and *Q* at the p-polarization are both larger than those at the s-polarization, and this also holds for FOM, as the quantity derived from them.

The regularities (i)–(iv) stated above, as well as the A-types of sensors’ characteristics variation with the change of their layers’ materials, could be understood, at least qualitatively, with analytical models, which have been developed in the past. Though some of these have been cited and discussed in Refs. [2,46], we extend the overview from the 1990s to the present. Given the vast number of papers on this issue, we do not claim the references list considered to be comprehensive. For GMR structures that embed rectangular lamellae gratings, the RCWA numerical modeling was reported [47] before any analytical theory. Later on, analytical treatments based on mergers of a zero-order approximation to RCWA with the planar-waveguide theory [48,49,50], those based on more involved equations obtained via RCWA truncation in the spirit of the Kogelnik coupled-mode theory [51], and those based on the latter while mimicking a surface-relief grating by one written inside a photo-refractive layer [52], were developed. Subsequently, the fabrication and characterization of GMR-based notch optical filters [53,54,55] and electro-optical modulators [51,56] were reported, showing a fair agreement between the experiment and theory. At that time, no suggestion for employing the GMR effect under external irradiation in optical sensing was proposed; thus, the *S* and FOM characteristics were not even considered.

An idea of joining a grating with a flat multilayer for sensing in the GMR-configuration was borrowed more than two decades ago from another, albeit related, field, namely sensors based on grating coupler (GC); see, e.g., overviews [57,58,59]. Although both GMR- and GC-based sensors utilize gratings, they are basically different in many other aspects. Contrary to the GMR structures, which typically involve a separate grating layer atop the waveguide, as considered above, the GC structures contain a grating with a mostly sawtooth profile incorporated directly into a patch of the waveguide surface. Fabricating such gratings requires a precise alignment between the grating and the waveguide, often involving embossing, multiple lithography steps, and replication [59,60,61,62].

GC sensors operate in the output or input modes (OM or IM). In the OM, e.g., [60], laser radiation fed into the waveguide via fiber, launches a guided mode. The mode passing through the grating region can, under appropriate conditions, fully out-couple the radiation off the sensor via the substrate as a diffracted plane wave with the propagation direction making an angle to the normal defined by the celebrated grating equation. In the IM, e.g., [61], the sensor is irradiated from the backside by a plane wave with the propagation directed at the same out-coupling angle, but on the other side of the normal. Diffracted by the grating, the impinged wave launches the guided mode, which is read out with a detector placed at the waveguide exit.

Thus, the GMR sensor shares similarities with the IM- or OM-GC sensor solely in terms of the types of light waves fed into or exiting the device, respectively, but the physics underlying their functionality and signal readout schemes are fairly different. In the GMR sensor, it is the optical resonance between the excited guided mode and the reflected (or transmitted) wave that drives the sharp device response, which is recorded by measuring the spectral angular polarized reflectance (or transmittance). The changes in the latter occur due to changes in the assay, while scanning over either λ or θ is operative for sensing. The physical quantities determined by the GC sensors are the effective RIs of the TE and TM modes supported by the single-mode waveguide, which change in response to changes in the analyte. For OM- or IM-GC sensors operating at fixed λ’s, the effective RIs directly depend on the exit or acceptance angles, respectively. Therefore, angular scanning is the only available method for GC sensing, which demonstrated a wide range of capabilities from RI sensing [63] to label-free techniques, such as immunological and affinity sensing [58,62,64,65] as well as biological and biochemical sensing [60,63,66], to which the GMR sensors are approaching these days.

For context, in addition to the recent research on the GMR sensors quoted earlier, original studies, including experimental ones, which have been reported to the best of our knowledge—see, e.g., review [67]—starting from the 2000th, are to be noted. In particular, Wawro et al. reported [68] an RCWA analysis, fabrication, and successful testing of a GMR sensor including a photoresist grating on a Si_3_N_4_ waveguide affixed to the end faces of an optical fiber. This served as the light input to and output from the GMR unit, in contrast to the GMR sensors responding to light incident from free space. The measured DOL of that sensor was $3.2\cdot 10^{-4}\;\mathrm{RIU}$ at a resolution of 0.1nm of the spectroscopic setup used, which corresponds to S=312.5nm/RIU. This sensitivity, maintained almost constantly over a wide span of 1.3–1.7 for the assay RI variation, supported the claim [68] that this is a versatile sensor with a large dynamic range. The SRU Biosystems (Woburn, MA, USA) and Nano Sensors (University of Illinois at Urbana-Champaign, Champaign, IL, USA) groups designed, manufactured, and analyzed GMR biosensors employing Si_3_N_4_ and TiO_2_ 1D gratings on polymer and porous glass substrates embossed in a cured epoxy [69] and a low-RI porous glass layer [70], respectively. Similar work was performed in SRU Biosystems [71] with concern to the GMR sensors using a 2D grating on a glass substrate, in which another glass filled the grooves and coated the surface.

The studies [69,70,71] carried out the design with early commercial versions of FDTD-based tools and Lumerical FDTD Solutions, and focused on sensing biological and biochemical species in a thin adsorbed assay layer. Rather than RI changes, these sensors detect the changes in layer thickness, which are recalculated with respect to the species density changes. A mass-density sensitivity resolution of $10^{-13}\;g/{\mathrm{mm}}^{2}$, and even finer with the choosing of proper materials, was reported. Later, research on the GMR sensors using diverse combinations of other suitable materials was published [72,73]. The sensor reported in Ref. [72] was numerically designed and simulated with the latest version of the original RCWA [28]. It comprised a glass substrate, 1.5μm and 450nm thick SiO_2_ lower-cladding and Si_3_N_4_ waveguide core layers, respectively, grown in sequence by a PECVD, and a grating with Λ=900nm fabricated atop the Si_3_N_4_ layer with molding and UV-NIL. Spectral and angular sensitivities of about 100–150 nm/RIU and 100°/RIU upon illumination under θ=0° by a laser tunable in the range of 1.46–1.58 μm and fixed λ=632.8nm laser source, under oblique incidence in the range of 50–80°, respectively, were obtained in transmission mode. In Ref. [73], the numerical design and simulations were performed with a freely distributed RCWA software package. Sensor samples were fabricated from commercial SOI substrates with a planar 220nm thick Si waveguide on a 3μm thick buried oxide layer. The SiO_2_ gratings with d=180nm and Λ=1.24μm atop the Si layer were obtained by electron beam lithography using a resist, which forms SiO_2_ upon electron beam exposure. The p-polarized reflectance spectrum of the laser light in a band of λ = 1.50–1.55 μm was measured at θ=45° fixed in the optical setup, and the value of S≃110nm/RIU, in agreement with theory, was reported.

The Swiss group, whose experiments on the GC sensors are cited above, also developed [74] an analytic theory of sensitivities of their response to changes in the assay RI and its thickness. That theory was adapted [75], including chromatic corrections to the effective RIs of the grating layer, to the GMR sensors, enabling the calculation and optimization of their sensitivity *S*. Semi-analytical computations, as confirmed by RCWA, showed [75] that *S* for $n_{\sup}>n_{\mathrm{sub}}$ and $n_{\sup}<n_{\mathrm{sub}}$, with our study matching the latter case, essentially differ. In Ref. [75], a GMR sensor embedding a grating with Λ=420nm and DC=0.5 etched to d=25nm in a thicker Si_3_N_4_ layer on a SiO_2_ substrate was optimized for sensing ethanol-in-water ambient at θ=0°, and the p-polarized sensitivity of S≃104nm/RIU was obtained at t=100nm. This sensor had *t* and Λ values very close to those of all our sensors, t=110nm and Λ=450nm, respectively; however, it has a shallower grating and differs in terms of materials and structural details. Ignoring for now the differences in *d* and $\lambda_{r}$, and given a closeness of RIs of our substrate and analyte to those of the corresponding constituents of the sensor treated in Ref. [75], the latter turns out to be the closest to our prototype A2. Despite that, the p-polarized sensitivity of A2 at θ=0°, 86nm/RIU, proves lower, but that of A1, S=102nm/RIU, is about the same, as seen in Table 1. This example demonstrates that the design of a high-sensitivity GMR sensor is achieved not only through the combination of dimensional and material parameters, but rather through their unique interplay. The latter, due to researchers’ consensus—see, e.g., [46,67] and references therein—is such that it provides maximal penetration of the waveguide-mode evanescent field into the assay.

The above factor *Q* of the GMR sensors has no obvious correlation with their *S*. But, as noted before, the increasingly high *Q*, i.e., decreasingly small Δλ, results in the GMR structures acquiring increasing FOM, which allows for more precise sensing at small Δλ; see, e.g., [1,46]. The main regularity of *Q*, learned from studies of the GMR optical filters with no sensing functionality in the past [47,48,49,50,51,52,53,54,55,56] and reviewed recently [1,2], applies to the GMR sensors on equal footing. Namely, the parameter that controls Δλ at θ=0° is the permittivity contrast between the grating groove and groove, which, in our configuration, equals $\Delta \varepsilon =n_{\mathrm{gr}}^{2}-n_{\sup}^{2}$; the smaller the Δε, the smaller the Δλ and hence a larger *Q*. The data of Table 1 plausibly confirm this rule. For example, the factor *Q* of the A5 sensor is significantly larger than that of the A3 sensor, which agrees with the fact that the RI index of PVC is much closer to the RI of the assay than the RI of CsPbBr_3_. For the same reason, a similar comparison sustains for the factors *Q* of the A4 and A1 sensors.

Normal-incidence configurations dominated the past research on GMR optical filters, and only a few papers considered an oblique incidence. These presented the dependence of Δλ on θ by formulas hardly usable for an analysis of simulations and experimental data. The same can be said about the most recent publications on the GMR sensors, which present their characteristics at θ=0°, in which *Q* ranges between 100 and 300 [1], whereas only a few works have published numerical simulations of the GMR sensors’ performance under an oblique incidence [11,45,76,77]. In particular, Guo et al. [11] tested a photoresist grating (photolithography fabrication) on Ta_2_O_3_. However, these studies have not presented systematic numerical testing of the *S* and *Q* versus θ.

According to Table 1, the CsPbBr_3_ grating on the Si_3_N_4_ waveguide structure (A3) shows the sensitivities *S* of 276nm/RIU and 440nm/RIU at the incidence angles of 30° and 60°, respectively, for the p-polarization. Notably, the fabrication of this grating does not require photolithography. In addition, the table showcases the PVC grating on Si_3_N_4_ waveguide sensor (A5) that achieves the p-polarized $Q≃4.01\cdot 10^{4}$ at θ=60°. As a result, this sensor’s unique FOM at p-polarization, viz. $\mathrm{FOM}≃1.36\cdot 10^{7}\;\mathrm{nm}/\mathrm{RIU}$, is significantly—by order of magnitude—larger than the values published previously. In particular, Liang et al. [78] and Zhou et al. [1] reported $Q≃10^{4}$, $\mathrm{FOM}≃7\cdot 10^{5}\;\mathrm{nm}/\mathrm{RIU}$, and $Q≃8\cdot 10^{3}$ and $\mathrm{FOM}≃1.08\cdot 10^{6}\;\mathrm{nm}/\mathrm{RIU}$, respectively, to mention a few. A more extensive citations list can be found in the review paper [46].

### 3.2. 2D Grating Sensor at Normal Incidence

**Figure 10:**
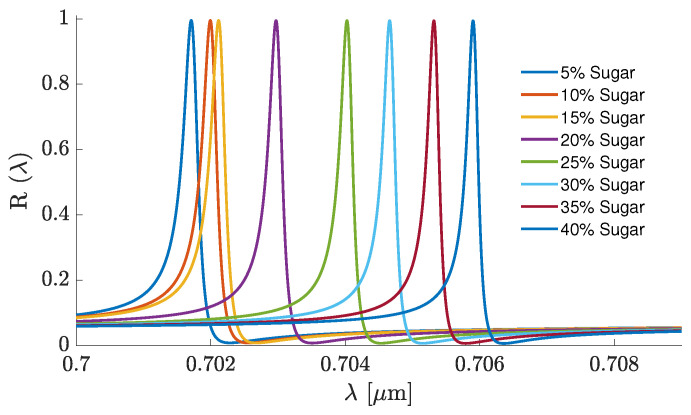
The normal-incidence reflectance spectra of the GMR sensor B2 at different sugar concentrations, which are shown in the legend.

The sensor structures based on a 2D grating on a Si_3_N_4_ waveguide were simulated for the normal incidence of light, as seen in Figure 2. The gratings’ materials employed in the RCWA simulations were Si_3_N_4_, PVC, and CsPbBr_3_. The simulated normal-reflectance spectra and benchmark characteristics of the selected B-type sensors are shown in Figure 10 and Table 2, respectively. Table 3 presents a comparison of characteristics for selected polarization-insensitive B- and polarization-sensitive A-type sensors.

As can be concluded from Table 2 and Table 3, using a 2D grating significantly improves the sensor benchmarks (*S*, *Q*, and FOM) as compared to the GMR sensors based on 1D gratings.

Although the detailed parametric analysis in this work focuses on 1D GMR structures under oblique incidence, we included 2D grating configurations at normal incidence to demonstrate the design flexibility and to highlight their promising performance characteristics. The observed improvements in *Q* and FOM suggest that 2D structures may offer enhanced sensing capabilities, and we plan to investigate their behavior under oblique incidence in future work.

## 4. Discussion

Unlike most previous studies that focused on normal incidence due to its straightforward implementation, this work systematically investigates the effect of oblique incidence on GMR sensor performance. The presented analysis of *Q* and FOM as functions of incidence angle provides new insights into achieving high-*Q* resonances without being constrained by nanofabrication limitations [79]. Recent studies have shown that oblique incidence can significantly enhance GMR sensor performance, improving sensitivity up to 2.9 times compared to normal incidence [11,80]. Furthermore, systematic analyses demonstrated that *Q* and FOM can reach record values at optimized angles [81], highlighting the importance of exploring angle-tuned configurations beyond the conventional normal-incidence approach [82].

Although the detailed parametric analysis in this study focused on 1D GMR structures under oblique incidence, including 2D grating configurations (at normal incidence) serves an essential purpose. First, it demonstrates the design flexibility of the proposed approach, showing that the proposed material systems and fabrication methods are compatible with both 1D and 2D geometries. Second, the observed improvements in *Q* and FOM for 2D structures—even without angle tuning—suggest that these configurations may offer superior sensing performance, motivating further investigation into 2D GMR sensors under oblique incidence, which is a study to pursue in future work.

Beyond the scope of guided-mode resonance sensors, recent studies have demonstrated the versatility of metasurface platforms in various photonic applications. For instance, adjustable slow light and optical switching have been achieved in black phosphorus metamaterials via double plasmon-induced transparency [83]. TiN-only metasurface absorbers have shown promise for solar energy harvesting [84]. Dirac semimetal-based metasurfaces have also enabled dynamic terahertz tuning [85] and tunable multi-band absorption and sensing [86]. These developments highlight the broader relevance of resonance-based metasurface designs, including GMR configurations, in emerging optical technologies.

## 5. Conclusions

Novel GMR sensors designed for glucose and hemoglobin concentration based on lead-halide perovskite and PVC sub-wavelength gratings are presented at normal and oblique incidence. In these designs, polymer and perovskite allow flexible, low-cost fabrication by soft-lithography/imprint-lithography methods. The sensitivity, FOM, and *Q* are presented for 1D and 2D optical grating structures. The 1D perovskite grating sensor shows a performance similar to the silicon nitride grating sensor, where in all-perovskite sensors, increasing the incident angle and the grating pitch improves the sensitivity, *Q*, and FOM. These findings confirm that both perovskite and silicon nitride can be effectively employed in GMR sensors, offering flexibility for practical implementation where material choice may depend on fabrication constraints and integration requirements [23,24]. The characteristics of the 2D grating sensor were compared to the 1D sensor at normal incidence. The 2D grating sensor structures significantly improve *Q* and FOM compared to 1D grating structures. An ultra-high *Q* of 50,000 is achieved for 1D PVC grating on silicon nitride waveguide at p-polarization and 75 degrees obliqued incidence, which is about one order of magnitude larger relative to the *Q* value at normal incidence. Our study shows that one can avoid the time-consuming prefabrication numerical optimization required for precise fabrication of the GMR and effectively tune the sensing parameters by simply varying the incidence angle. Nonetheless, practical implementation may be influenced by factors such as material stability and the absence of experimental validation, which should be considered when evaluating the applicability of the proposed designs.

## Figures and Tables

**Table 1:** Concurrent impact of the materials and incidence angle on the benchmark characteristics of the set A sensors. The results presented in the table were computed for the reflectance peak wavelengths $\lambda_{r}$ up to a maximum of 1.1μm.

Set A structures, s-polarized sensing modality									
Structure	*S*, nm/RIU			*Q*			FOM, nm/RIU		
	0°	30°	60°	0°	30°	60°	0°	30°	60°
A1 ^∗^	59	287	– ^†^	$1.72\cdot 10^{2}$	$7.75\cdot 10^{2}$	– ^†^	$1.02\cdot 10^{4}$	$2.23\cdot 10^{5}$	– ^†^
A2 ^⋄^	55	277	441	$1.70\cdot 10^{2}$	$7.33\cdot 10^{2}$	$6.32\cdot 10^{2}$	$9.35\cdot 10^{3}$	$2.03\cdot 10^{5}$	$2.79\cdot 10^{5}$
A3 ^⋄^	56	276	441	$1.46\cdot 10^{2}$	$6.75\cdot 10^{2}$	$5.91\cdot 10^{2}$	$8.18\cdot 10^{3}$	$1.86\cdot 10^{5}$	$2.61\cdot 10^{5}$
A4 ^∗^	30	222	– ^†^	$3.83\cdot 10^{3}$	$1.35\cdot 10^{4}$	– ^†^	$1.14\cdot 10^{5}$	$3.00\cdot 10^{6}$	– ^†^
A5 ^⋄^	30	216	355	$2.16\cdot 10^{3}$	$8.40\cdot 10^{3}$	$8.64\cdot 10^{2}$	$6.48\cdot 10^{4}$	$1.82\cdot 10^{6}$	$2.89\cdot 10^{6}$
Set A structures, p-polarized sensing modality									
A1 ^∗^	102	306	– ^†^	$6.52\cdot 10^{2}$	$4.22\cdot 10^{3}$	– ^†^	$6.66\cdot 10^{4}$	$1.29\cdot 10^{6}$	– ^†^
A2 ^⋄^	86	275	401	$6.41\cdot 10^{2}$	$4.72\cdot 10^{3}$	$1.62\cdot 10^{4}$	$5.51\cdot 10^{4}$	$1.30\cdot 10^{6}$	$6.51\cdot 10^{6}$
A3 ^⋄^	88	276	402	$5.45\cdot 10^{2}$	$4.38\cdot 10^{3}$	$1.45\cdot 10^{4}$	$4.80\cdot 10^{4}$	$1.21\cdot 10^{6}$	$5.82\cdot 10^{6}$
A4 ^∗^	62	243	– ^†^	$8.91\cdot 10^{3}$	$3.21\cdot 10^{4}$	– ^†^	$5.53\cdot 10^{5}$	$7.81\cdot 10^{6}$	– ^†^
A5 ^⋄^	55	225	338	$5.61\cdot 10^{3}$	$2.57\cdot 10^{4}$	$4.01\cdot 10^{4}$	$3.09\cdot 10^{5}$	$5.79\cdot 10^{6}$	$1.36\cdot 10^{7}$

^†^ For these prototypes, at θ=60°, the GMR peak is out of the above-noted simulation range. ^∗^ The simulations were performed for the hemoglobin-in-water solution assay. ^⋄^ The simulations were performed for the glucose-in-water solution assay.

**Table 2:** Normal-incidence characteristics of type-B sensors, where the analyte was sugar solution.

Sensor Structure	S,nm/RIU	*Q*	FOM,nm/RIU
B1	82	$2.60\cdot 10^{3}$	$2.13\cdot 10^{5}$
B2	83	$2.44\cdot 10^{3}$	$2.03\cdot 10^{5}$
B3	67	$2.32\cdot 10^{4}$	$1.55\cdot 10^{6}$

**Table 3:** Comparison of 2D grating sensors to 1D grating sensors at normal incidence. The materials used for the structures are PVC for the gratings and Si_3_N_4_ for the waveguide. The ambient assay was the same as in Table 2.

Sensor Type	S,nm/RIU	*Q*	FOM,nm/RIU
A5: s-polarized	30	$2.16\cdot 10^{3}$	$6.48\cdot 10^{4}$
A5: p-polarized	54	$5.61\cdot 10^{3}$	$3.03\cdot 10^{5}$
B3	67	$2.32\cdot 10^{4}$	$1.55\cdot 10^{6}$

## Data Availability

The original contributions presented in this study are included in the article. Further inquiries can be directed to the corresponding authors.

## Abbreviations

The following abbreviations are used in this manuscript:

GMR	Guided-mode resonance
DPN	Dip-pen nanolithography
RCWA	Rigorous coupled-wave analysis
FOM	Figure of merit
NIL	Nanoimprint lithography
PECVD	Plasma-enhanced chemical vapor deposition
SOI	Silicon on insulator
